# A Novel Sphingan Containing Enriched Guluronic Acid Residues and Its Hydrogel Properties

**DOI:** 10.3390/polym18111339

**Published:** 2026-05-28

**Authors:** Haolin Huang, Yuanjun Lu, Weiyi Tao, Caifeng Li, Junzhang Lin, Shuang Li

**Affiliations:** 1 College of Biotechnology and Pharmaceutical Engineering, Nanjing Tech University, Nanjing 211816, China; 202262118011@njtech.edu.cn (H.H.); 202461218175@njtech.edu.cn (Y.L.); 2 College of Food Science and Light Industry, Nanjing Tech University, Nanjing 211816, China; taoweiyi@njtech.edu.cn; 3 Research Institute of Petroleum Engineering and Technology, Shengli Oilfield Company, Sinopec, Dongying 257000, China; licaifeng136.slyt@sinopec.com (C.L.); linjunzhang.slyt@sinopec.com (J.L.)

**Keywords:** biopolymers, hydrogel, sphingan, *Sphingomonas*, rheological properties

## Abstract

In this study, it is shown that the strain *Sphingomonas* sp. HL-1 can utilize glucose, xylose and mixed sugars to produce high yields (28.7 g/L) of HL gum, which has a high molecular weight of 2.03 × 10^6^ Da. HL gum is a novel sphingan uniquely enriched in guluronic acid (16.74%) and galacturonic acid, with low rhamnose content, distinguishing it from conventional sphingans. It exhibits good solubility in water at 25 °C and 60 °C, pronounced shear-thinning behavior, and concentration-dependent, heat-activated gelation. Remarkably, even at a low concentration of 1% (*w*/*v*), HL gum forms a resilient hydrogel upon heating to 90 °C, achieving a gel strength of 2950 ± 130 g/cm^2^, and maintains gel stability under high salinity (up to 50 g/L). SEM micrographs revealed a clear temperature-dependent morphological transition: from fibrous structures at 20 °C to curled aggregates at 60 °C and finally to a porous fibrous network at 90 °C, consistent with a sol–gel transition. Combined with its ability to efficiently utilize mixed sugars in a short fermentation cycle, HL gum’s unique chemical composition and superior gelation properties distinguish it from conventional sphingans, highlighting its potential for food, biomedical, and industrial hydrogel applications.

## 1. Introduction

Microbial polysaccharide is a polymer formed by the secretion and metabolism of microorganisms, and the main component is D-glucose [1]. It has the characteristics of short production cycle, green and environmental sustainability, and consistent yield [2] and is widely used in food, medicine, petroleum and other fields [3]. In microbial polysaccharides, sphingans represent a class of acidic extracellular polysaccharides produced by bacteria of the genus *Sphingomonas*. Notable examples include gellan, welan, diutan, and sanxan, each characterized by distinct molecular structures and rheological properties [4]. These polymers are valued for their ability to modify the rheology of aqueous systems, serving as effective gelling, thickening, stabilizing, and suspension agents [5].

Among sphingans, gellan gum and sanxan have been reported to have gelation properties. Gellan gum has the typical structure of sphingans because there is no glycosyl on the side chain, and its gel properties have also been widely studied [6]. Gellan gum dissolves well in hot water and forms a gel when the solution cools [7]. Sanxan is an atypical sphingan that lacks the tetrasaccharide unit module characteristic of typical sphingans, and it has similar gel properties [8]. Although only two exopolysaccharides with gel-forming properties produced by *Sphingomonas* have been discovered so far, both of them undergo conformation transformation with the stimulation of cations, thus forming gels [6,9,10]. The rheological properties of polysaccharides were also affected by cations [11].

In addition to gellan and sanxan among sphingans, curdlan represents another common polysaccharide exhibiting gel-forming properties, and its characteristics have been extensively studied. Unlike gellan gum, curdlan forms gels with varying strengths depending on the cooling temperature. Specifically, curdlan can form two distinct types of gels: a softer, thermoreversible “cold-set gel” when heated to approximately 55–60 °C followed by cooling, and a firmer, thermally irreversible “heat-set gel” when heated above 80 °C [12]. X.Man et al. [13] employed AFM and SEM to observe the molecular aggregation of curdlan under heating conditions, revealing that the curdlan bundles of triple-stranded helixes would gradually dissociate into single helical chains with the increase in temperature. This structural reorganization underpins curdlan’s capacity to produce heat-stable gels with high water-holding capacity and exceptional thermal stability. Consequently, the well-characterized gelation behavior and functional versatility of curdlan serve as a valuable reference for the development and application of novel sphingans.

Compared with conventional sphingans such as gellan and sanxan, HL gum is distinguished by its high content of guluronic and galacturonic acids, its low rhamnose content, and its ability to form strong hydrogels at low concentrations upon heating. Unlike ion-dependent gels, HL gum forms thermally stable hydrogels independent of cations. Furthermore, it can be efficiently produced from glucose, xylose, or their mixtures in a short fermentation period with high yields. These structural and functional advantages suggest HL gum as a versatile candidate for advanced hydrogel applications, including food structuring, biomedical hydrogels, and industrial processes.

## 2. Experimental Methods

### 2.1. Microorganism and Media

*Sphingomonas* sp. HL-1 used in the study was deposited at the China Center for Type Culture Collection (CCTCC NO: M2021162). The seed medium contained 20 g/L glucose, 1 g/L yeast extract, 2.33 g/L (NH_4_)_2_HPO_4_, 1 g/L K_2_HPO_4_·3H_2_O, 0.1 g/L MgSO_4_·7H_2_O, and 0.003 g/L FeSO_4_·7H_2_O at pH 7.0–7.2. The fermentation medium comprised 40 g/L sugar, 1 g/L yeast extract, 2.33 g/L (NH_4_)_2_HPO_4_, 1 g/L K_2_HPO_4_·3H_2_O, 0.1 g/L MgSO_4_·7H_2_O, and 0.003 g/L FeSO_4_·7H_2_O. The initial pH was adjusted to 7.0–7.2.

### 2.2. Fermentation of HL Gum

*Sphingomonas* sp. HL-1 was first inoculated into a 250 mL volumetric flask containing 50 mL seed medium and cultured continuously for 24 h at 30 °C. The batch production of HL gum was carried out in a 5 L stirred-tank bioreactor (ALPHA, Suzhou, China) at 30 °C. Seed culture (10%, *v*/*v*) was inoculated into the fermenter containing 3 L fermentation medium. The aeration rate was maintained at the level of 1 vvm, and the control of agitation speed varied from 300 to 1000 rpm. During the fermentation process, the pH of the fermentation broth was automatically controlled at 7.5 with a 2 M NaOH solution. The apparent viscosity of the fermentation broth was measured at 30 °C with a rotational viscometer (IKA, Staufenim, Germany) at 30 rpm with spindle 4. The optical density (OD) of the culture was measured using a spectrophotometer at 600 nm. Residual glucose was determined using an SBA-40E biosensor analyzer after appropriate dilution of the fermentation broth. Residual xylose was measured using a D-xylose assay kit (Beijing Solarbio Science & Technology Co., Ltd., Beijing, China).

### 2.3. Preparation of HL Gum

HL gum was extracted and purified via ethanol precipitation, a well-established method for polysaccharide isolation [14]. Briefly, the viscous fermentation broth was diluted 1:10 (*v*/*v*) with distilled water and subjected to heat treatment at 60 °C for 30 min. Subsequently, the mixture was centrifuged (10,000× *g*, 15 min, 4 °C) to remove microbial cells. The supernatant was concentrated under reduced pressure, and three volumes of cold 95% (*v*/*v*) ethanol were added. The mixture was incubated overnight at 4 °C, followed by centrifugation (8000× *g*, 20 min, 4 °C) to pellet the crude polysaccharide fraction. The precipitate was redissolved in preheated deionized water (60 °C) to ensure complete swelling then treated with an equal volume of Sevage reagent (chloroform:butanol = 5:1, *v*/*v*) to eliminate residual proteins. After phase separation and centrifugation, the aqueous phase was collected and dialyzed against distilled water using a 10,000 Da molecular weight cutoff membrane for 48 h (with buffer changes every 12 h). Finally, the dialysate was lyophilized to yield purified HL gum.

### 2.4. Observation of Bacterial Morphology by TEM

For transmission electron microscopy (TEM), HL-1 bacterial cells were fixed overnight at 4 °C with 2.5% glutaraldehyde, post-fixed with 1% osmium tetroxide, and dehydrated through a graded ethanol series. After acetone transition, samples were infiltrated with epoxy resin, embedded, and polymerized at 70 °C.

Samples prepared for TEM analysis were sectioned using a Leica EM UC7 ultramicrotome to obtain ultrathin sections (70–90 nm). The sections were then stained with lead citrate and uranyl acetate in a 50% ethanol saturated solution for 5–10 min each and subsequently observed under a Hitachi HT-7700 (Hitachi, Japan) transmission electron microscope.

### 2.5. Characteristics Analysis of HL Gum

#### 2.5.1. Molecular Weight (M_w_)

The M_w_ (weight-average molar mass), M_n_ (number-average molecular weight) and molecular mass dispersity (M_w_/M_n_) of the filtered HL gum solution (3 mg/mL) were analyzed using Waters 2695 HPLC (with a 2410 oscillometric refractive detector and Empower workstation). The column used was an Ultrahydrogel^TM^ linear gel filtration column (7.8 × 300 mm) (Waters Corporation, Milford, MA, USA). The detection conditions were set as 40 °C, 0.5 mL/min, and the mobile phase was 0.1 M sodium nitrate solution.

#### 2.5.2. The Monosaccharide Composition of HL Gum

About 5 mg of purified HL gum was weighed precisely and placed in an ampoule. Then, 1 mL of 2 M TFA (trifluoroacetic acid) was added, and the mixture was kept at 121 °C for 2 h for complete hydrolysis. Subsequently, the solution was dried under a nitrogen stream. Methanol was added for washing, followed by drying again; this methanol washing step was repeated 2–3 times. Finally, the residue was dissolved in sterile water and transferred into a chromatographic vial for analysis. The supernatant was characterized by IC (ionic chromatography) analysis. The sample was loaded on an ICS5000 system (Thermo Fisher Scientific, Waltham, MA, USA) equipped with a DionexCarbopacTMPA20 column at 30 °C [15]. Data were acquired on the ICS5000 (Thermo Scientific, Waltham, MA, USA) and processed using chromeleon 7.2 CDS (Thermo Scientific).

A mixture of 13 monosaccharide standards (fucose, rhamnose, arabinose, galactose, glucose, xylose, mannose, fructose, ribose, galacturonic acid, glucuronic acid, guluronic acid, and mannuronic acid) was prepared according to the method described above. Quantitative analysis was performed using an absolute quantification method, and the molar ratios of the monosaccharides were calculated based on their respective molar masses.

#### 2.5.3. Fourier Transformed Infrared (FT-IR) Spectroscopy

The purified HL gum was pressed into transparent film. FT-IR spectra of sample were recorded by a Nicolet spectrometer from 4000 to 400 cm^−1^ [8].

### 2.6. Rheological Properties

#### 2.6.1. Concentration Selection

The concentration range of 0.075–0.175% (*w*/*v*) was selected based on previous studies on sphingan-type polysaccharides (e.g., welan, xanthan, and gellan systems), particularly those investigating their rheological behavior in dilute to semi-dilute regimes. This range also ensures that the system remains below the concentration required for the formation of a fully developed stable gel, while allowing observation of concentration-dependent changes in viscoelastic properties [16].

For salinity experiments, a fixed HL gum concentration of 0.075% (*w*/*v*) was used, as this threshold concentration provides the highest sensitivity to the enhancing or disruptive effects of ions on the nascent gel network.

#### 2.6.2. Steady Shear Measurement

HL gum was dissolved in ultrapure water and configured into aqueous solutions of different concentrations (0.075–0.175%, *w*/*v*). The solution was placed at 60 °C and completely swollen. The apparent viscosity of the above samples was measured by a DHR-2 rheometer (TA Instruments, New Castle, DE, USA) with a 40 mm parallel plate fixture. Viscosity data were collected at shear rates from 0.01 s^−1^ to 100 s^−1^ at 25 °C, and Origin was used to process data (OriginLab Corporation, Northampton, MA, USA).

#### 2.6.3. Dynamic Viscoelastic Behavior

The viscoelastic properties (gelation behavior) of HL gum were investigated by oscillatory rheometry under varying concentrations, salinities, and temperature. First, HL gum was dissolved in ultrapure water to prepare aqueous solutions at different concentrations (0.075–0.175%, *w*/*v*). The solutions were fully swollen at 60 °C. Their dynamic viscoelastic behavior was then measured at 25 °C within a frequency range of 0.1–1.0 Hz while maintaining a constant strain of 1%.

HL gum was dissolved in brines of different salinities to a final concentration of 0.075% (*w*/*v*). The dynamic viscoelastic behavior of the mixture was similarly measured at 25 °C under the same frequency and strain conditions. The composition of the brine water containing mixed metal ions is presented in Table 1.

Finally, temperature sweep measurements were performed on HL gum solutions (1% and 2%, *w*/*v*) across a temperature range of 20–85 °C at a fixed frequency of 6.28 rad/s and a constant strain of 1%.

### 2.7. Microstructure of HL Gum Imaged Using SEM

HL gum solution (0.5 mg/mL) was first prepared by dispersing purified HL gum powder (0.01 g, dry basis) into 20 mL ultrapure water in a vial bottle, with stirring using a WH220-HT digital stirrer (Wiggens, Wertheim, Germany) at 400 rpm until the powder was completely dissolved at 20 °C. Samples were then incubated at 20, 60 and 90 °C separately, with stirring at 400 rpm for 2 h. The HL gum aqueous dispersion was quickly quenched with liquid nitrogen, placed in a refrigerator at −80 °C for 2 h, and then placed in a freeze dryer. The samples obtained were used for SEM tests.

Samples required by SEM needed to be gold-sprayed before the experiment, and so the prepared sample was glued to the sample table with a conductive adhesive. The samples were observed using a field emission scanning electron microscope (Quanta FEG 250, FEI Company, Hillsboro, OR, USA) at an accelerating voltage of 10 kV. Images with magnifications of 3000× and 6000× were recorded.

### 2.8. Gel Strength Measurement

The pure HL gum was dissolved in deionized water (2%, *w*/*v*) and fully swollen in a water bath at 50 °C, 70 °C and 90 °C for 30 min; then, the morphological of HL gel was observed after cooling to room temperature.

To evaluate the strength of gels formed by HL gum, the following procedure was conducted: the HL gum aqueous solution was placed in a mold with a diameter of 9.5 mm, aerated under vacuum for 3 min, and then rapidly placed in a water bath at 90 °C for 30 min. After cooling to room temperature, the gel was removed from the mold for subsequent testing. The gel was tested by a compression test on a CMT2103 microcomputer control electronic universal testing machine (MTS, Shenzhen, China), and the gel strength (*W*) was calculated by

(1) $$Gel\;strength\;(W)=\;F/g/\pi r^{2}$$

where F represents the reading of the inflection point in the load–time (*F*-*T*) curve at which the curve drops sharply when the gel breaks, expressed in newtons (N); g is the acceleration of gravity in meters per second squared (m/s^2^); and r is the radius of the mold, in millimeters (mm).

## 3. Results and Discussion

### 3.1. Production Performance of HL Gum

**Figure 1:**
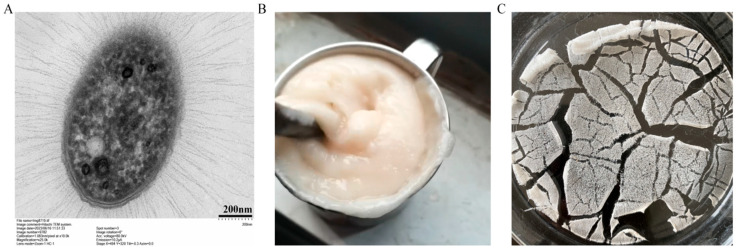
(**A**) TEM image of strain HL-1, (**B**) the viscous fermentation broth resulted from gel production by the HL-1 strain, and (**C**) lyophilized powder of purified HL gum.

The strain *Sphingomonas* sp. HL-1 was a Gram-negative, rod-shaped bacterium, with cells measuring approximately 1 μm in length and 0.5 μm in width (Figure 1A). Strain HL-1 formed smooth, circular colonies with a raised elevation on agar plates, exhibiting an opaque and milky white appearance. When cultivated at 30 °C with glucose as the sole carbon source, strain HL-1 demonstrated efficient production of extracellular polysaccharides. In contrast to typical sphingan-producing strains, which commonly synthesize yellow carotenoid pigments [17], strain HL-1 failed to produce these pigments. Consequently, its fermentation broth exhibited a distinctive milky-white appearance and markedly elevated viscosity, as illustrated in Figure 1B. Following extraction and purification, the HL gum extract appeared as a white, fluffy powder (Figure 1C), which was subsequently subjected to structural characterization.

**Figure 2:**
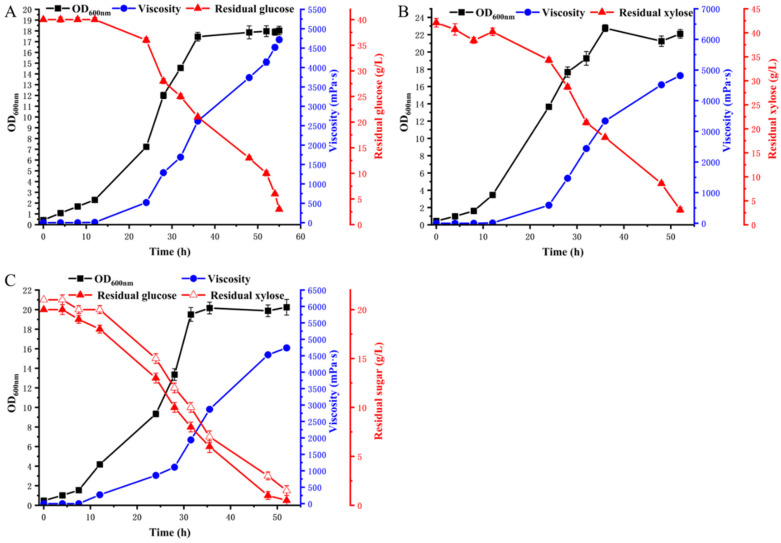
Time course of HL gum production by the strain *Sphingomonas* sp. HL-1 ((**A**): glucose, (**B**): xylose, (**C**): 1:1 glucose-xylose).

In our previous study, strain HL-1 produced 20 g/L of HL gum in shake-flask cultures [18]. In the present work, we systematically evaluated its polymer production capacity in a 5 L bioreactor using glucose, xylose, and a glucose–xylose mixture as carbon sources. Key process parameters—including residual reducing sugar concentration, HL gum titer, and broth apparent viscosity—were monitored throughout fermentation. As shown in Figure 2A,B, fermentations with glucose and xylose yielded 28.7 g/L and 28.0 g/L of HL gum in 55 h and 52 h, respectively. Under both conditions, residual reducing sugar declined to ≤3 g/L and apparent viscosity reached 4720 mPa·s and 4820 mPa·s, respectively. These results demonstrate that both glucose and xylose can serve as effective carbon sources for the production of HL gum. Notably, when an equimolar mixture of glucose and xylose was used as the carbon source (Figure 2C), strain HL-1 exhibited the capacity to co-utilize both sugars simultaneously. The fermentation period was maintained at 52 h, with the apparent viscosity and HL gum titer reaching 4740 mPa·s and 28.2 g/L, respectively. The ability of strain HL-1 to concurrently utilize glucose and xylose for gel production closely resembles the metabolic behavior of strain NX02 [19], which simultaneously assimilates these sugars to biosynthesize a novel sphingan named Sanxan. Among sphingans, gellan gum and rhamsan gum have not been reported to be synthesized using xylose as a carbon source. Liu et al. [20] adopted a two-step fermentation strategy for welan gum production, wherein glucose mother liquor (GML) was used to promote cell growth, followed by xylose mother liquor (XML) for gum synthesis. Importantly, strain HL-1 retains the ability to co-metabolize xylose even in the presence of high glucose concentrations, suggesting the absence of carbon catabolite repression (CCR) [21]. This distinctive metabolic trait enables the direct use of lignocellulosic hydrolysates—complex substrates rich in mixed sugars—for efficient gel fermentation.

The gel production capability of biopolymer-producing strains in laboratory-scale bioreactors typically serves as a key indicator for assessing their potential for commercial-scale viability. The production of sphingans is influenced by multiple factors, including carbon and nitrogen sources, their concentrations, temperature, and pH [22,23]. The typical sphingan-producing strains and their respective production capabilities are summarized in Table 2. Currently, the yields of most sphingans range from 20 to 30 g/L. Among them, strain HL-1 exhibits the advantages of high polysaccharide yield and short fermentation duration, with significant advantages in production performance.

### 3.2. Monosaccharide Composition and Structural Characteristics of HL Gum

The weight-average molecular weight (M_w_) and number-average molecular weight (M_n_) of HL gum were determined to be 2.03 × 10^6^ Da and 5.93 × 10^5^ Da, respectively. The dispersity index (M_w_/M_n_) was calculated to be 3.42, which indicates a relatively broad molecular weight distribution. The monosaccharide composition of HL gum, as detailed in Table 3, reveals its distinctive polymeric structure. Monosaccharide composition analysis (Figure S1) demonstrated that HL gum is primarily composed of glucose (72.89%) and guluronic acid (16.74%), with minor constituents including mannose, galactose, ribose, and several uronic acids. The monosaccharide composition analysis indicates that HL gum is an acidic heteropolysaccharide.

The uronic acid components of HL gum synthesized in different culture systems exhibit significant differences. Notably, the guluronic acid content in HL gum (16.74%) is significantly higher than that reported in our previous study (2.5%), in which sucrose was used as the sole carbon source [18]. Specifically, the remarkable increase in the guluronic acid component is potentially associated with variations in nutritional components of the culture medium, such as differences in carbon sources and nitrogen sources or changes in culture conditions including pH and dissolved oxygen. These combined nutritional and environmental factors may have synergistically contributed to the observed alteration in polysaccharide composition.

Typical sphingans such as gellan, welan and sanxan are usually composed of glucose, rhamnose, mannose and glucuronic acid as the main components [30]. As shown in Table 3, a distinctive characteristic of HL gum is its remarkably low content of rhamnose components and the presence of abundant guluronic acid residues. While guluronic acid is relatively uncommon in microbial polysaccharides, it constitutes a principal structural unit of alginate, which is a linear anionic polysaccharide composed of β-D-mannuronate (M) and α-L-guluronate (G) residues arranged in M, G, and MG blocks. In alginate, the G blocks form junction zones upon binding divalent cations (Ca^2+^) via the “egg-box” model, which governs its ionic cross-linking gelation behavior. This compositional profile distinguishes HL gum from conventional sphingans and aligns it more closely with alginate, highlighting the significance of guluronic acid content and sequence for gel formation and mechanical properties [31]. In alginate, the content and sequential arrangement of guluronic acid are critical determinants of its rheological behavior, gelation capacity, and mechanical strength—properties that underpin its broad applications in drug delivery, immunomodulation, and tissue engineering [32]. Therefore, we subsequently conducted a more in-depth rheological characterization of HL gum and placed particular emphasis on its gelation properties.

### 3.3. FT-IR Spectra Analysis of HL Gum

**Figure 3:**
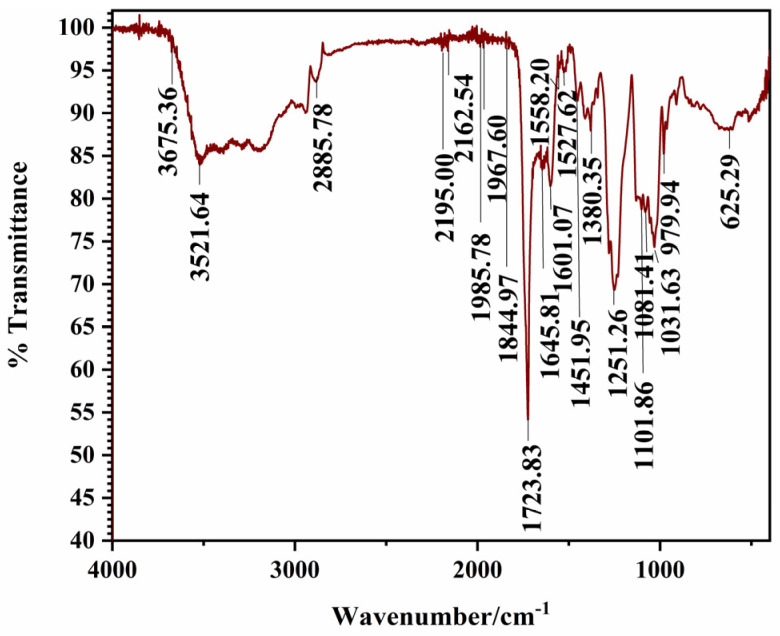
FT-IR spectra of HL gum.

Fourier-transform infrared (FT-IR) spectroscopic analysis of purified HL gum (Figure 3) revealed that the band at 3675.36 cm^−1^ was the characteristic absorption peak of free O-H, and the band at 3521.64 cm^−1^ was caused by the stretching vibration of polysaccharide O-H, which was the characteristic peak of polysaccharide. The band of 2885.78 cm^−1^ was caused by the stretching vibration of glycidyl C-H, and the weak bands near 1723.83 cm^−1^ and 1645.81 cm^−1^ were assigned to the C=O stretching vibration of protonated carboxyl groups and the asymmetric stretching vibration of carboxylate groups (COO^−^), respectively, which are characteristic absorption bands of uronic acid-containing acidic polysaccharides, indicating that HL gum was an acidic polysaccharide [33,34]. The bands in the 1558.20 cm^−1^, 1451.95 cm^−1^ and 1380.35 cm^−1^ regions were caused by C-H or O-H bending vibration, while 1251.26 cm^−1^ was caused by O-H deformation vibration. The bands between 1101.86 cm^−1^, 1081.41 cm^−1^ and 1031.63 cm^−1^ were characteristic absorption peaks of pyranose ring C-O-C [34]. In addition, the band at 979.94 cm^−1^ indicated the presence of β-type glycosidic linkage in HL gum [8]. These FT-IR results further confirmed that HL gum was an acidic polysaccharide with a pyranose ring and β-type glycosidic linkage.

### 3.4. Rheological Properties of HL Gum

Rheological properties such as shear-thinning behavior, viscoelasticity, and ion-responsive gelation are central to unlocking the application potential of sphingans. These properties dictate their performance across diverse fields, from food and pharmaceuticals to oil recovery and tissue engineering.

#### 3.4.1. Solubility and Shear Response

To assess the industrial applicability of HL gum, a polysaccharide within this family, we initiated a systematic investigation of its rheological behavior, beginning with its fundamental solution properties and shear response.

As a prerequisite for rheological characterization, the solubility of HL gum was first evaluated. The HL gum can achieve complete swelling in pure water at 25 °C, and dissolution accelerated markedly at 60 °C, typically completing within 30 min due to thermally enhanced molecular motion [35]. This confirmed its suitability for preparing homogeneous aqueous solutions for subsequent analysis.

**Figure 4:**
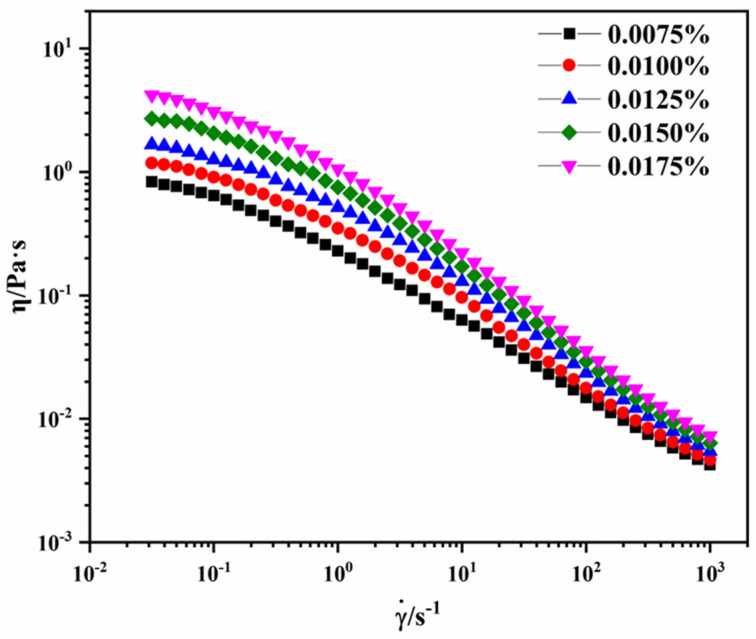
Shear response of HL gum at different concentrations (0.075–0.175%; *w*/*v*).

The shear-dependent viscosity, a key rheological parameter, was then examined (Figure 4). Across a concentration range of 0.075–0.175%, all HL gum solutions exhibited consistent shear-thinning behavior—a marked decrease in viscosity with an increasing shear rate. This behavior aligns with the typical response of random coil polysaccharides [36] and provides an initial insight into its flow behavior under applied stress.

#### 3.4.2. Dynamic Viscoelastic Behavior on Concentrations of HL Gum

To characterize the viscoelastic properties of hydrogels, which are defined as water-rich, polymer-based networks exhibiting elastic solid-like behavior, we performed dynamic oscillatory rheometry. The energy storage modulus (G′) and the loss modulus (G″), also known as the elastic modulus and the viscous modulus, represent the elastic and viscous nature of the material. Frequency sweep experiments were conducted to evaluate the structural integrity and molecular dynamics of the hydrogel across different concentrations, where changes in G′ and G″ with frequency reflect the underlying network architecture and entanglement [37].

**Figure 5:**
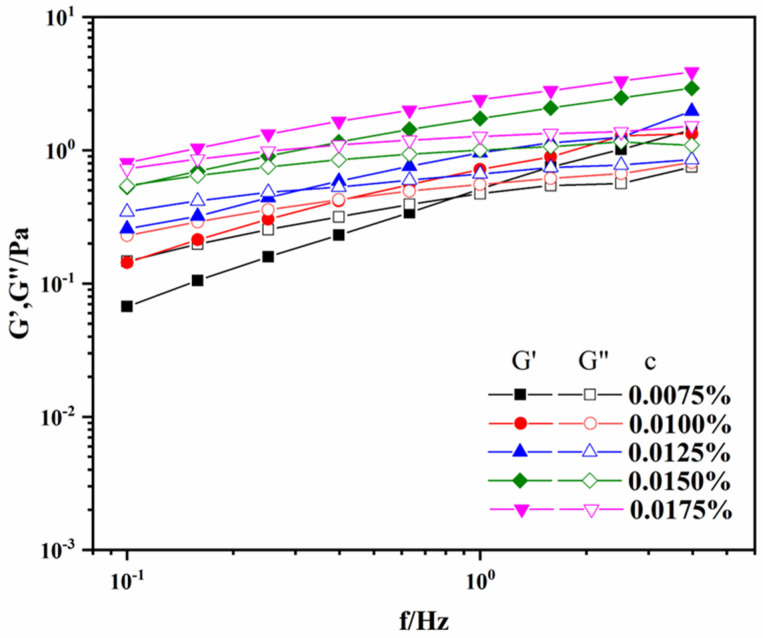
Dynamic viscoelastic behavior of HL gum at different concentrations (0.075–0.175%; *w*/*v*).

As can be seen in Figure 5, the rheological behavior of HL gum was strongly concentration-dependent within the tested range (0.075–0.175%, *w*/*v*). With increasing concentration, the frequency range where G′ exceeded G″ expanded, indicating a shift from a predominantly viscous fluid (G″ > G′) toward a more elastic, gel-like state (G′ > G″). At low concentrations, the system behaved as a viscoelastic fluid at low frequencies but showed a progressive transition to solid-like behavior at higher frequencies, implying the formation of transient or frequency-induced network structures. At the highest concentration (0.175%), G′ consistently surpassed G″ across the entire frequency spectrum (ranging from ≈1–10 Pa), confirming the establishment of a continuous gel network. This evolution in mechanical response, transitioning from fluid-like to weak gel-like behavior, reflects the concentration-dependent and frequency-dependent structural reinforcement of HL gum, consistent with the general characteristics of physical hydrogels where network strength increases with polymer content. Similar concentration-dependent gelation behavior has been reported for other sphingans, such as sanxan, which forms gels at concentrations above 0.1% (*w*/*v*) [10].

The frequency-dependent behavior of the dynamic moduli, G′ and G″, was modeled using a power-law relationship described by G′(*ω*) = *Aω^a^* and G″(*ω*) = *Bω^b^*, where *A* and *B* represent the consistency coefficients for the elastic and viscous components, respectively, while *a* and *b* denote their corresponding frequency exponents, reflecting the structural stability and shear-thinning characteristics of the material. The power-law parameters obtained from dynamic frequency sweeps (Table 4) reveal that both elastic (*A*) and viscous (*B*) coefficients increase with HL gum concentration, while the frequency exponents (*a*, *b*) decrease.

The elastic coefficient *A* increases consistently with concentration, from 0.500 Pa at 0.075% to 2.306 Pa at 0.175%, indicating a strengthened gel network and enhanced elastic response at higher concentrations. The frequency exponent *a* decreased from 0.766 to 0.391 as the concentration rose, showing that G′ becomes less frequency-dependent. At 0.175%, *a* ≈ 0.39, suggesting that the material behaves more like a stable, solid-like gel (weak frequency dependence of elasticity). The viscous coefficient *B* also rose with concentration (0.437 Pa to 1.222 Pa), reflecting increased energy dissipation during deformation, consistent with a denser polymer network. The exponent *b* decreased from 0.382 to 0.172, indicating that G″ becomes nearly frequency-independent at high concentrations, which is typical for gels where viscous flow is suppressed by network formation. Across all concentrations, *A* > *B*, confirming that elastic behavior dominates over viscous behavior (G′ > G″), characteristic of a weak gel. The decrease in both *a* and *b* with increasing concentration demonstrates that the system evolves from a more fluid-like, frequency-sensitive state (0.075%) toward a more solid-like, network-controlled gel (0.175%).

As shown in Table 4, a stable three-dimensional network forms, leading to solid-like rheological responses. This concentration-dependent sol–gel transition is consistent with the behavior of many physical hydrogels, where chain entanglement and intermolecular interactions become significant beyond a critical polymer content.

#### 3.4.3. Dynamic Viscoelastic Behavior on Salinity of HL Gum

**Figure 6:**
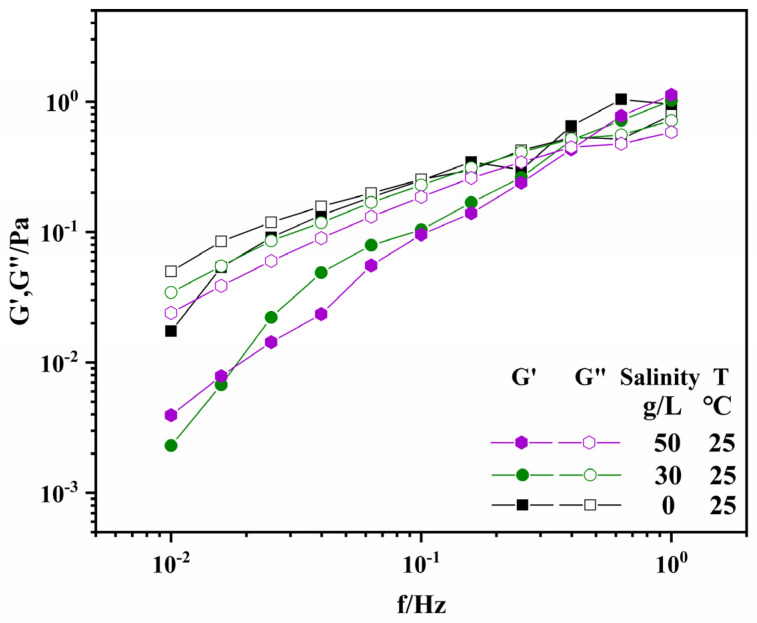
Dynamic viscoelastic behavior of HL gum (0.075%, *w*/*v*) at different salinities.

The dynamic frequency sweep results clearly demonstrate the inherent gel-forming capability of the HL gum, independent of salinity induction. To investigate the dependence of the viscoelastic behavior of HL gum on salinity, the effects of Na^+^, Ca^2+^, and Mg^2+^ on G′ and G″ were examined. Different levels of salinity (via mixed metal ions) were introduced into HL aqueous solutions (0.075%, *w*/*v*), and the results are presented in Figure 6.

At a constant temperature of 25 °C, the HL gum solution exhibited distinct weak gel-like behavior in the absence of added ions (0 g/L salinity). Upon the addition of ions, the moduli of HL gum, particularly G″, decreased notably. When the mixed ion concentration reached 50 g/L, further reductions in both G′ and G″ were observed. The decline in absolute modulus values reflects a softening effect, likely due to electrostatic screening or competitive interactions partially disrupting the native polymer associations [38]. Concurrently, the crossover point of G′ and G″ in the HL hydrogel shifted slightly toward higher frequencies. This indicates that the presence of metal ions inclined the system toward a more sol-like state, suggesting that the viscoelastic behavior of HL gum is not positively dependent on metal ions and their addition does not promote the formation of a stronger HL hydrogel network.

These results reveal a key functional distinction of HL gum. Unlike ion-gelling biopolymers such as alginate [39], HL gum is capable of establishing a stable hydrogel network through non-ionic, physical interactions. This salt-independent yet ion-tunable behavior positions HL gum as a robust gelling agent, especially suitable for applications where ionic concentration may vary or where consistent gelation must be maintained in low-salt or salt-free conditions.

Sodium alginate is a classical ion-sensitive polysaccharide, and its gelation is strongly dependent on the presence of divalent cations. For example, 3 wt% alginate solutions show a rapid sol–gel transition upon the addition of 7.5–15 mg/L Ca^2+^, with the elastic modulus G′ rapidly increasing and exceeding G″ by approximately one order of magnitude and reaching plateau values within tens of seconds [40]. Higher Ca^2+^ concentrations lead to faster gelation and higher plateau moduli.

In contrast, HL gum forms weak gel-like networks without any added divalent cations. Time sweep experiments across a wide range of salinities (0–50 g/L) show only gradual changes in G′ and G″, with the crossover point shifting slightly to higher frequencies, but no abrupt sol–gel transition is observed (Figure 6). These results indicate that HL gum gelation is governed predominantly by non-ionic interactions, such as chain entanglement and hydrogen bonding. The comparison clearly demonstrates the ion-independent sol–gel transition of HL gum versus the rapid, ion-dependent gelation of alginate.

#### 3.4.4. Dynamic Temperature Behavior of HL Gum

**Figure 7:**
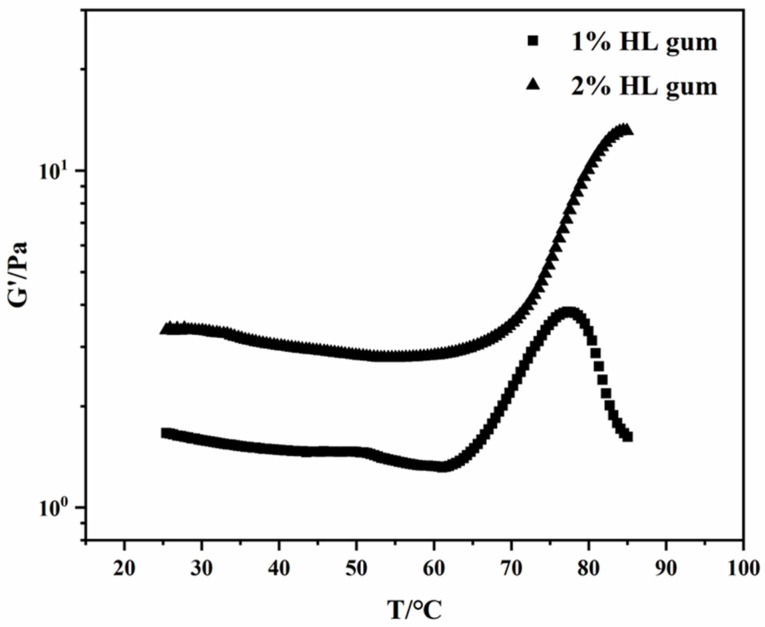
Temperature dependence of G′ for HL gum (1% and 2%, *w*/*v*).

The gelation behavior of HL gum was investigated using thermal scanning rheological measurements. To elucidate its nature as a heat-set gel and its concentration-dependent activation, we specifically compared a dilute system (1%) near the gelling threshold with a concentrated system (2%) representing robust gel formation. The dynamic storage modulus (G′) as a function of temperature was recorded for 1% and 2% aqueous suspensions of HL gum during heating (Figure 7). The profiles for both concentrations are characteristic of a heat-activated gelation process, where a temperature increase provides the necessary energy to disrupt initial associative structures and facilitate the reorganization into a stable network.

For the 1% HL gum solution, the G′ began to increase at approximately 60 °C, which may be attributed to the thermal disruption of intermolecular hydrogen bonds. This process enhances chain mobility and promotes the molecular rearrangement and aggregation of polysaccharide chains. As temperature further increases, these rearranged chains associate to form a more stable and continuous three-dimensional network structure, leading to an increase in elasticity [41]. The G′ reached a maximum around 80 °C, indicating that the network formation and structural stabilization were maximized at this temperature, which may be attributed to the thermal disruption of intermolecular hydrogen bonds [35]. For the 2% HL gum aqueous solution, the G′ began to increase at approximately 70 °C and continued to rise with temperature, reaching a maximum around 85 °C. The consistent increase in G′ beyond 60 °C indicates the formation of a heat-activated gel.

The distinct concentration-dependent onset temperatures highlight that the heat-set trigger is not an absolute value but a system property modulated by polymer concentration. The data demonstrate that HL gum exhibits classic heat-activated gelation, where concentration critically governs both the thermal activation point and the ultimate rheological properties of the resulting gel. This provides a fundamental framework for tailoring its functionality in thermal processing applications.

### 3.5. Microstructure of HL Gum

The thermal gelation behavior of HL gum exhibits characteristics of a classic heat-activated process, sharing mechanistic similarities with well-documented gelling polysaccharides like curdlan, which forms irreversible gels upon heating above a critical temperature [42]. Given that curdlan’s gelation is a paradigmatic example of heat-set gelation driven by conformational changes and chain aggregation upon thermal activation, it serves as an excellent reference model. To elucidate the microstructural evolution underlying the heat-activated gelation of HL gum, we employed scanning electron microscopy (SEM) to examine samples after thermal treatments. The microstructure of HL gum samples treated at three key temperatures (20 °C, 60 °C, and 90 °C) was observed by SEM. These temperatures were selected to capture the microstructural states corresponding to the pre-activation, activation/initiation, and post-activation/network maturation stages of the heat-set process.

**Figure 8:**
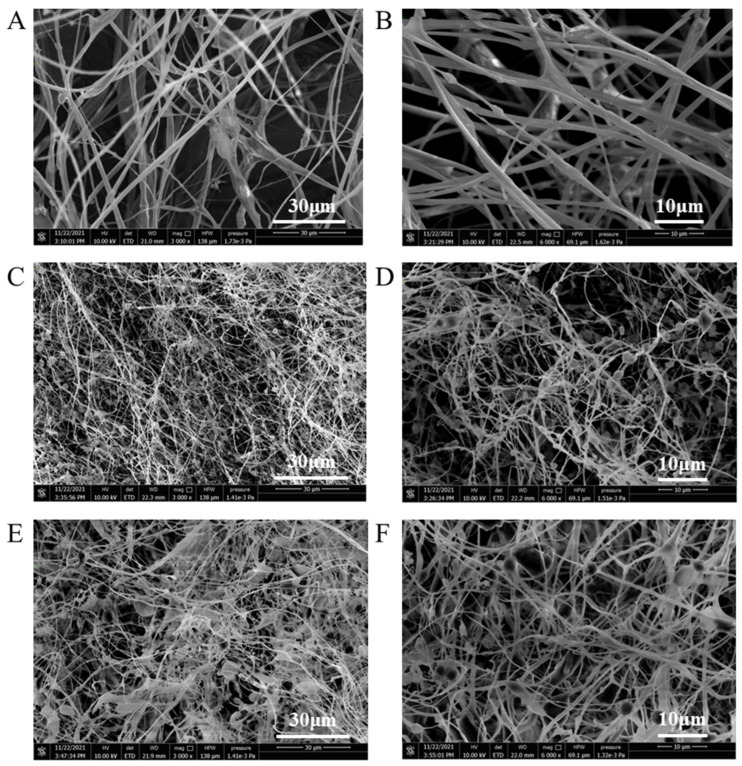
SEM (scanning electron microscopy) micrographs presenting surface morphology of HL gum at 20 °C (**A**,**B**), 60 °C (**C**,**D**) and 90 °C (**E**,**F**). The magnifications of each group images are set as 3000× and 6000×, respectively.

The microstructure of HL gum changed significantly in the temperature range of 20–90 °C. In the initial state of HL gum (20 °C), it was observed that HL gum showed a coarse and dispersed fibrous structure with relatively regular overall arrangement, large space between fibers, and a small amount of swelling in the middle of fibers (Figure 8A,B). When the sample temperature was raised to 60 °C, the fiber width was reduced and the winding was more obvious, showing a state of irregular crimping, and the interfiber space was also reduced. At the same time, there were a certain amount of fiber aggregates at the fiber end (Figure 8C,D). When the sample treatment temperature reached 90 °C, the fibers became finer, the crimped structures appeared relaxed, and the fiber aggregates expanded to form a porous fibrous network (Figure 8E,F).

These morphological changes indicate that HL gum undergoes temperature-dependent structural transitions. Interestingly, this thermally induced morphological evolution resembles the behavior of curdlan, a well-characterized polysaccharide that forms heat-set gels via temperature-dependent conformational changes [43,44]. Based on this similarity, we speculate that HL gum may undergo a comparable process: a regular fibrous microstructure at room temperature, followed by apparent structural rearrangement upon heating. However, direct evidence at the molecular level is needed to confirm this hypothesis.

### 3.6. Thermal Gelation Performance and Gel Strength of HL Gum

**Figure 9:**
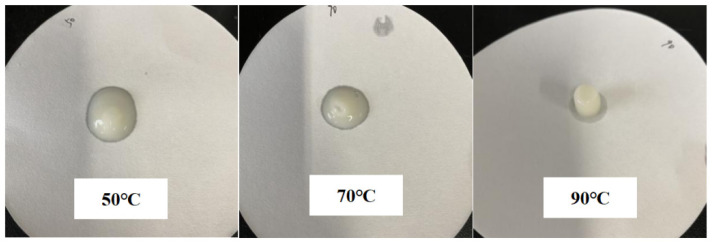
Effect of thermal treatment on the morphology of HL solution (2%, *w*/*v*). The diameter of the droplets was approximately 9.5 mm.

The gelation process of HL gum was further characterized by monitoring its macroscopic state after controlled thermal treatments. The morphological changes of the solutions (2%, *w*/*v*) were subsequently evaluated, as shown in Figure 9. The solution was insufficient to form a gel with a stable morphology after treatment at 50 °C and 70 °C for 30 min. However, a resilient, freestanding hydrogel formed upon heating to 90 °C, a temperature well above the activation threshold, followed by cooling. This indicates that HL gum undergoes a heat-activated sol–gel transition, where a critical thermal energy input is necessary to trigger permanent network assembly.

The resulting HL hydrogel (2%, *w*/*v*) exhibited a gel strength of 3140 ± 150 g/cm^2^, classifying it as a high-strength gel. Notably, this robust gelation is concentration-efficient, as even a 1% (*w*/*v*) solution formed a high-strength gel (2950 ± 130 g/cm^2^) after the same thermal activation at 90 °C. This reinforces the conclusion that HL gum is a potent, heat-set gelling agent whose functionality is initiated by surpassing a critical thermal threshold.

The gel strength of HL gum was compared with that of curdlan, a microbial polysaccharide known for forming high-strength, thermally irreversible gels. Curdlan is a linear β-(1→3)-glucan composed exclusively of glucose residues, which forms gel networks through the formation of triple-helix structures upon heating [45]. Gao et al. [27] reported that curdlan produced by the wild-type strain *Agrobacterium* sp. ATCC 31,479 had a molecular weight of 8.47 × 10^5^ g/mol and exhibited a gel strength of 1736 g/cm^2^ at the concentration of 4% (*w*/*v*). HL gum has a molecular weight of 2.03 × 10^6^ g/mol, which is significantly higher than that of curdlan. Notably, at a significantly lower concentration of 1% (*w*/*v*), HL gum achieved a remarkably high gel strength of 2951.68 g/cm^2^—approximately 1.7 times greater than that of curdlan at 4% (*w*/*v*). This result highlights that the superior gel-forming efficiency of HL gum under dilute conditions is closely related to its high molecular weight.

The differences in gel performance can be partly attributed to the distinct solubility and gelation mechanisms among polysaccharides, as summarized in Table 5. While gellan gum requires thermal dissolution and sets upon cooling and curdlan exhibits thermo-gelling behavior that depends on heating conditions, sanxan gum and alginate show good cold-water solubility and gel primarily via ionic cross-linking. Unlike the above, HL gum exhibits a concentration-dependent, heat-activated gelation characterized by a well-defined thermal threshold. This comparative analysis not only contextualizes the unique rheological behavior of HL gum within the broader landscape of microbial and plant-based gums but also highlights its potential as a novel thermo-stable gelling agent. Future research should focus on elucidating its precise molecular structure, optimizing its gel properties for specific applications (e.g., food texture modification or biomedical hydrogels), and exploring its synergistic effects with other biopolymers.

## 4. Conclusions

This study demonstrates that HL gum, produced by *Sphingomonas* sp. HL-1, is a novel sphingan with unique structural and functional properties. It is primarily composed of glucose and guluronic/galacturonic acids, exhibits excellent water solubility, and forms stable hydrogels through concentration-dependent, heat-activated gelation. Even at 1% (*w*/*v*), HL gum forms high-strength gels (2950 ± 130 g/cm^2^) and maintains gel integrity under high-salinity conditions (up to 50 g/L). SEM observations confirmed a temperature-dependent microstructural evolution, from fibrous networks to curled aggregates and finally to porous fibrous networks, consistent with the observed rheological transitions. Compared with conventional microbial polysaccharides such as gellan and curdlan, HL gum offers superior low-concentration gelation efficiency, high molecular weight, and non-ion-dependent gelation, positioning it as a versatile biomaterial with broad potential in food structuring, biomedical hydrogels, and industrial applications.

## Figures and Tables

**Table 1:** Composition of the brine water.

Composition (g/L)			Total Salinity (g/L)
NaCl	CaCl_2_	MgCl_2_	
22.7	2.70	4.55	30
37.9	4.50	7.58	50

**Table 2:** Typical sphingan-producing strains and their production capacities.

Gum	Strains	Carbon Source	Nitrogen Source	Time (h)	Yield (g/L)	Reference
HL	*Sphingomonas* sp. HL-1	Glucose 40 g/L	Yeast extract 1 g/L, (NH_4_)_2_HPO_4_ 2.33 g/L	55	28.7	This work
Gellan	*Sphingomonas paucimobilis* ATCC 31461	Sucrose 40 g/L	Monosodium glutamate 1.25 g/L	48	27.86	[24]
Gellan	*Sphingomonas paucimobilis* ATCC 31461	Sucrose 20 g/L	Soybean meal 1.9 g/L	62.2	7.55	[23]
Welan	*Alcaligenes* sp. CGMCC2428	Glucose 50 g/L	(NH_4_)_2_SO_4_ 8 g/L	72	24.9	[25]
Sanxan	*Sphingomonas sanxanigenes* NX02	Glucose 40 g/L	Peptone 5 g/L	80	21.20	[26]
Curdlan	*Agrobacterium* sp. ATCC 31749	Glucose 50 g/L	Yeast extract 1	80	20.82	[27]

**Table 3:** Monosaccharide composition of HL gum and representative sphingans.

Sphingans	Carbon Source		Monosaccharide Composition (mol %)							References
		Glucose	Mannose	Rhamnose	Galactose	Arabinose	Guluronic Acid	Galacturonic Acid	Glucuronic Acid	
HL	Glucose	72.89	3.78	0.41	1.37	0.19	16.74	2.49	0.25	This work
HL	Sucrose	89.3	1.9	-	0.4	0.2	2.5	5.5	-	[18]
Gellan	Sucrose	56.9	-	27.7	-	-	-	-	15.4	[28]
Welan	Glycerol	47.31	12.37	24.92	-	-	-	-	15.04	[29]
Sanxan	Sucrose	52.95	35.29	5.88	-	-	-	-	5.88	[8]

**Table 4:** Power-law fitting parameters of the dynamic moduli G′ and G″ for HL gum hydrogels at different concentrations.

Concentration (%, *w*/*v*)	*A* (Pa·s *^a^*)	*a*	*B* (Pa·s *^b^*)	*b*
0.075	0.500	0.766	0.437	0.382
0.100	0.694	0.531	0.533	0.299
0.125	0.901	0.523	0.644	0.223
0.150	1.668	0.423	0.948	0.174
0.175	2.306	0.391	1.222	0.172

**Table 5:** Comparative monosaccharide composition of HL gum and representative sphingans.

Gum	Solubility	Properties	Micro-Network Structure	Reference
HL	Fully soluble at 25 °C; dissolution accelerated at 60 °C.	(1) Concentration-dependent, heat-activated gelation (≥70–90 °C). (2) Gel forms upon heating, independent of cations. (3) High-strength gel at low concentrations (2950 ± 130 g/cm^2^ at 1%, *w*/*v*). (4) Tolerant to high salinity (up to 50 g/L).	Regular fibrous structure at 20 °C; irregular curled chains at 60 °C; porous sheet-like network at 90 °C.	This work
Gellan	Requires heating to ≧70 °C for complete dissolution in water.	(1) Gel forms upon cooling to 40 °C. (2) Gel strength enhanced by cations, especially divalent ions.	Coil-to-helix transition of macromolecular chains during gelation.	[7,30]
Sanxan	Fully soluble at 25 °C; dissolution rate increased at 60 °C.	(1) Gel forms readily in aqueous solutions above 0.1% concentration. (2) Enhanced by cations, especially Ca^2+^.	Mainly cyclic configurations composed of side-by-side intermolecular associations, with many rounded nodes found.	[10,19]
Curdlan	(1) Insoluble in cold water; forms dispersion at 55–60 °C. (2) Dissolved in aqueous NaOH and dimethyl sulphoxide (DMSO).	Two types of gels formed depending on heating temperature (thermoreversible gel at 55–60 °C, thermoirreversible gel at ≥80 °C).	Triple-stranded helices separate at 25–50 °C; partially dissociate at 60–70 °C; further dissociate into single strands at 80–90 °C.	[13,27]
Alginate	Soluble in cold (25 °C) and hot water.	(1) Ion-induced gelation (e.g., Ca^2+^) at 25–60 °C. (2) Gel strength depends on G-block content and sequence.	“Egg-box” model dimerization between guluronate blocks cross-linked by divalent cations.	[32,46]

## Data Availability

The original contributions presented in this study are included in the article/Supplementary Material. Further inquiries can be directed to the corresponding author.

## Supplementary Materials

The following supporting information can be downloaded at https://www.mdpi.com/article/10.3390/polym18111339/s1, Figure S1. Ion chromatography profiles of standard monosaccharides and HL gum.
