# Supplementary material for: A Novel Sphingan Containing Enriched Guluronic Acid Residues and Its Hydrogel Properties
Source: Polymers (Basel). 2026 May 28;18(11):1339. doi: 10.3390/polym18111339 (PMC13258888; doi:10.3390/polym18111339)
Supplement: Supplementary file 1 [file polymers-18-01339-s001.zip › polymers-4323752-supplementary.pdf]

Supplementary Materials

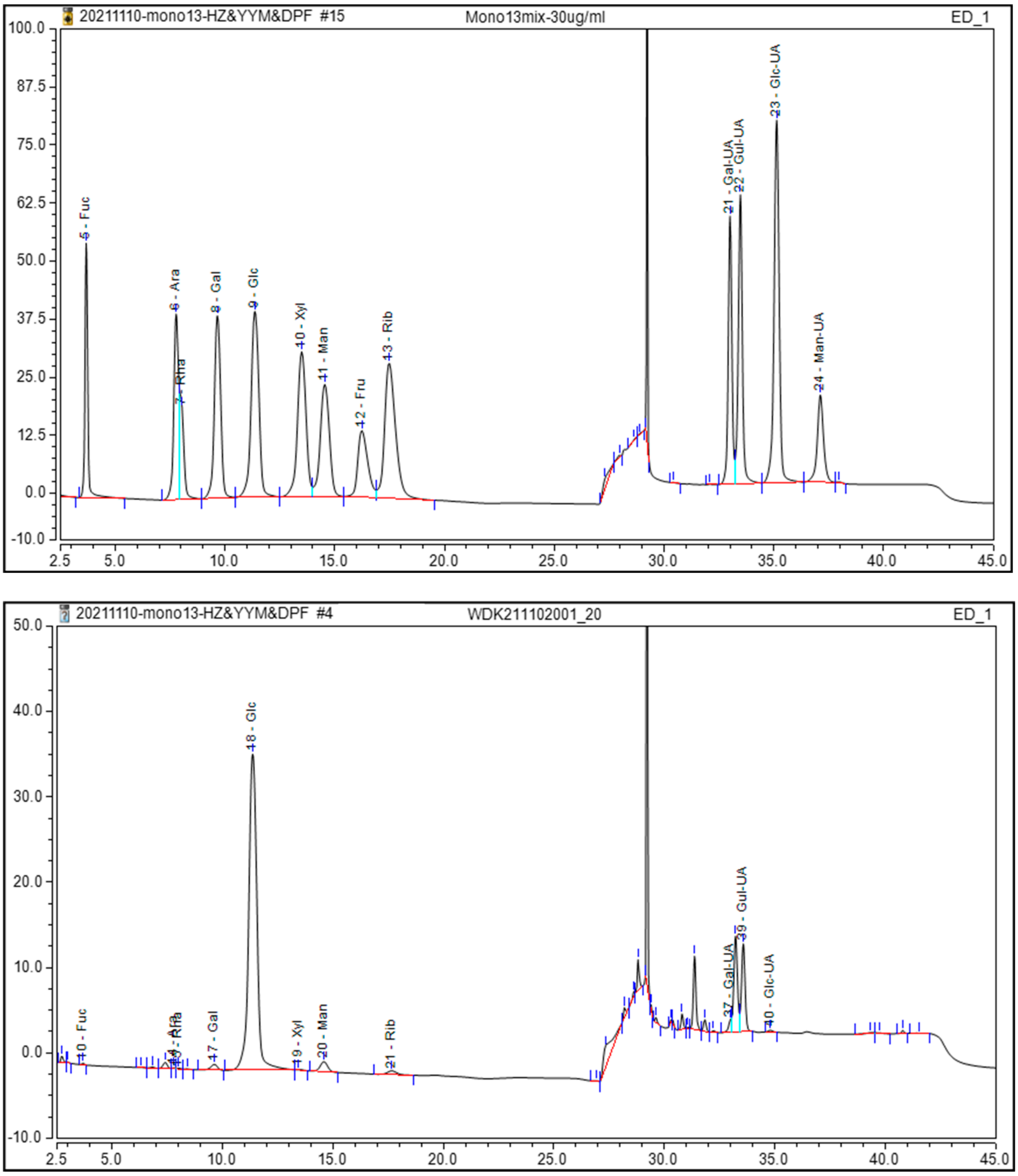

Figure S1. Ion chromatography profiles of standard monosaccharides and HL gum.
